# Supplementary material for: Extending beyond individual caves: a graph theory approach broadening conservation priorities in Amazon iron ore caves
Source: PeerJ. 2024 Jan 31;12:e16877. doi: 10.7717/peerj.16877 (PMC10838110; doi:10.7717/peerj.16877)
Supplement: Supplemental Information 2 — The type, unit, concept (description), code and method applied to the obtained data for each variable are informed. [file peerj-12-16877-s002.docx]

|  | Group | Variable | Type | Unit | Description | Code | Sampling method |
| --- | --- | --- | --- | --- | --- | --- | --- |
| Landscape features | Location and cave insertion | Longitude | Continuous | m | Longitudinal location | UTM_E | GPS Garmin 62s |
|  |  | Latitude | Continuous | m | Latitudinal location | UTM_N | GPS Garmin 62s |
|  |  | Altitude | Continuous | m a.s.l | Vertical distance from the sea level | Alt | GPS Garmin 62s |
|  |  | Scarp height | Continuous | m | Height of the steep slope (superior to 49°) in which the cave is inserted | Esc | GPS Garmin 62s |
|  |  | Geomorphological compartment | Categorical | - | Landscape regions with distinct physiographic traits | Gcomp | Classification based in three sectors^1^: summit, shoulder or footslope |
|  | Climate data | Vapour pressure | Continuous | kPa | Atmospheric pressure exerted on the surface | Vapr | WorldClim Data 2.0 (Fick & Hijmans, 2017) |
|  |  | Maximum temperature | Continuous | ^o^C | Average epigean maximum temperature | Tmax | WorldClim Data 2.0 (Fick & Hijmans, 2017) |
|  |  | Minimum temperature | Continuous | ^o^C | Average epigean minimum temperature | Tmin | WorldClim Data 2.0 (Fick & Hijmans, 2017) |
|  |  | Average temperature | Continuous | ^o^C | Average epigean temperature | Tavg | WorldClim Data 2.0 (Fick & Hijmans, 2017) |
|  |  | Solar radiation | Continuous | kJ m^-2^ day^-1^ | Average solar radiation on the surface | Srad | WorldClim Data 2.0 (Fick & Hijmans, 2017) |
|  |  | Precipitation | Continuous | mm | Average precipitation on the surface | Prec | WorldClim Data 2.0 (Fick & Hijmans, 2017) |
|  | Vegetation | Forest cover | Continuous | ha | Area covered by ombrophile forest in a 250 m buffer from the cave entrance geographical coordinates | ForC | QGis 3.10 (QGIS 2020) |
|  |  | Savannah cover | Continuous | ha | Area covered by metallophilic savannah in a 250 m buffer from the cave entrance geographical coordinates | CngC | QGis 3.10 (QGIS 2020) |
| Cave features | Physical features | Horizontal projection | Continuous | m | Sum of conduit lengths along the cave central axis | Hp | Obtained by reviewing the topographic maps^2^ |
|  |  | Depth | Continuous | m | Altimetric difference between the most elevated and the lowest ground level of the cave | Dpt | Obtained by reviewing the topographic maps^2^ |
|  |  | Area | Continuous | m^2^ | Area projected in the cave floor plan over the horizontal projection | Area | Obtained by reviewing the topographic maps^2^ |
|  |  | Volume | Continuous | m^3^ | Product of multiplying the area by the average height of conduits | Vol | Obtained by reviewing the topographic maps^2^ |
|  |  | Planimetric pattern | Categorical | - | Spatial morphology in the floor plan | Plan | Obtained by reviewing the topographic maps^2^ and the characterization of planimetric patterns adapted from Calux et al. (2019) |
|  |  | Granulometry | Categorical | - | Predominant size of substrate over the floor | Gran | Wentworth scale^3^ |
|  |  | Small-scale morphological features | Discrete | Types | Distinct types of morphological features formed by erosion/dissolution | Smf | Types based on the Lundberg (2012) approach^4^ |
|  | Lithology | Lithotypes | Categorical | - | Type of matrix rock: canga formations (CNG), banded iron formations (BIF), the combination of these two (B+C), or other less common lithotypes (OTHER). | Litho | Evaluation of composition, texture, color, structure, and weathering state of the outcrops within the caves. Lithotypes nomemclature followed Piló et al. (2015) |
|  | Trophic characteristics | Bat guano | Continuous | m^2^ | Area of feces deposition by bats over the cave floor | BatG | The perimeter of each deposit was delimited by positioning the pile vertices in the topographic map, so that the sum of areas occupied by such piles could be obtained through AutoCAD LT^®^ platform |
|  |  | Vertebrate traces | Categorical | - | Vestiges (like feces, carcasses and regurgitation) indicating the presence/use by vertebrates (except Chiroptera) | FzC | Visual observation of feces, carcasses, and regurgitation in the caves |
|  | Climatic factors | Maximum temperature | Continuous | ^o^C | Maximum temperature measured during sampling events | MaxT | Thermohygrometer^5^ |
|  |  | Minimum temperature | Continuous | ^o^C | Minimum temperature measured during sampling events | MinT | Thermohygrometer^5^ |
|  |  | Maximum humidity | Continuous | % RH | Maximum humidity measured during sampling events | MaxH | Thermohygrometer^5^ |
|  |  | Minimun humidity | Continuous | % RH | Minimum humidity measured during sampling events | MinH | Thermohygrometer^5^ |
|  |  | Hydric features | Categorical | - | Occurrence of hydric activity | Wt | Presence was recorded during the field works without defining their types (for example, drainage, pool, subterranean lake). Classification based in two conditions: perennial or intermittent. |

**^1^**Adapted from hillslope positions indicated by Miller and Schaetzl (2015). **^2^**Maps elaborated by Fundação Casa de Cultura de Marabá (accuracy 5D, according to British Cave Research Association – BCRA) (Scherer et al. 2019). **^3^**Characterization of planimetric patterns in the Appendix Table A1. **^4^**Clay (<0.004 mm), silt (0.004–0.064 mm), sand (0.064–2 mm), granule (2–4 mm), pebble (4–64 mm), cobble (64–256 mm) and boulder (>256 mm). **^4^**Types of small-scale morphological features in the Appendix Table A2. **^5^**Inconterm (model 7429.02.0.00, accuracy ± 1 ºC, ± 5% UR).

**References**

**Fick SE, Hijmans RJ. 2017.** WorldClim 2: new 1-km spatial resolution climate surfaces for global land areas. *International Journal of Climatology* **37(12)**:4302–4315 DOI 10.1002/joc.5086.

**QGIS Development Team. 2020.** QGIS geographic information system. Open source geospatial foundation project. *Available at http://qgis.osgeo.org*.

**Scherer BS, Scherer RS, Silva TM. 2019.** Levantamento espeleológico na Serra do Tarzan, Parauapebas, Pará. Revista Sumaúma, 11, 135-144. *Available at http://casadaculturademaraba. org/wp-content/uploads/2019/12/Revista-Sumau%CC%81ma-FCCM.2019.pdf* (accessed 06 August 2020).
