# Supplementary material for: Extending beyond individual caves: a graph theory approach broadening conservation priorities in Amazon iron ore caves
Source: PeerJ. 2024 Jan 31;12:e16877. doi: 10.7717/peerj.16877 (PMC10838110; doi:10.7717/peerj.16877)
Supplement: Supplemental Information 3 [file peerj-12-16877-s003.docx]

| Planimetric pattern | Planimetric types | Features | Code |
| --- | --- | --- | --- |
| Simple chamber | Rectangular | Approximately right angle in general controlled by fractures or other structure | SC_Rc |
|  | Funnel-shaped | Tappering in the distal direction, acquiring a funnel shape | SC_Fn |
|  | Semicircular | Approximately semi-circular shape | SC_Sc |
|  | Bifurcated | Divided into two branches | SC_Bf |
|  | Rectilinear | Follows a singles Direction in general conditioned by fractures or inclined plane of the rock banding | SC_Rt |
|  | Curvilinear | Curvatures present in the development axis, which may be conditioned to the junction between different fracture plans or even contact between the fracture plan and banding | SC_Cv |
|  | Misshapen | The configuration does not resemble any plane geometric form | SC_Ms |
| Compound chamber | Spongework | Conduits with variable sizes in general interconnected by narrow passages | CC_Sp |
|  | Mixed | Presence of more than one configuration | CC_Mx |
|  | Misshapen | The configuration does not resemble any plane geometric form | CC_Ms |
