# Supplementary material for: Extending beyond individual caves: a graph theory approach broadening conservation priorities in Amazon iron ore caves
Source: PeerJ. 2024 Jan 31;12:e16877. doi: 10.7717/peerj.16877 (PMC10838110; doi:10.7717/peerj.16877)
Supplement: Supplemental Information 4 [file peerj-12-16877-s004.docx]

| Morphological features | Description | Probable genesis | Code |
| --- | --- | --- | --- |
| Pillar | Elongated features with longer vertical projection formed in jaspilite and canga connecting the ceiling to the cave floor. They represent relic structures of old conduit walls that had intercepted. | Differential erosion of the rock massif that may be related to the material resistance and/or geological structures such as fractures and sub-vertical failures. | A |
| Pendant | Features with approximately conic morphology projected from the ceiling, with base narrower than top. | Formed by the differential dissolution of the surrounding iron rock. Pendants in ferruginous caves may be related to the erosion of old pillars. | B |
| Rocky arch | Elongated features with longer horizontal projection connected in both extremities to the conduit walls. They are relic structures formed in canga, jaspilite and mafic and represent the old base level of conduits. | Differential erosion of the rock massif that may be related to the material resistance and/or geological structures such as fractures and sub-horizontal failures. | C |
| Rocky step | Rocky step in the conduit floor or wall. This structure marks the old base level and differs from tiered level since it is not suspended. | Differential erosion of the rock massif that may be related to fractures, failures, mineral cover and/or differences on the composition and resistance of the rocky massif. | D |
| Tiered level | Suspended fragment of encrusted floor in general supported by iron oxyhydroxides, sulfate and phosphate covers in ferruginous caves. These features mark old base levels of conduits. | Differential erosion of the rock massif that may be related to fractures, failures, mineral cover and/or differences on the composition and resistance of the rocky massif. | E |
| Drip pits | Holes in the rocky floor with centimetric to decimetric length, in general circular or elliptical, formed under concentrated dripping on the outcrop rock. | They are formed by the water action in environments with greater concentration of guano and/or abrasion of clastic fragments on the floor of ferruginous caves. | F |
| Ferruginous voids | Funnel-like channels (curved or not) in general with centimetric diameter, that may develop both in the conduit floors or ceilings. | They are predominantly erosive features in ferruginous caves generated by the material removal during massif alterations that may be conditioned by geological structures or rock composition differences. | G |
| Ceiling cupola | Conic holes with sub-rounded base developed in the conduit ceiling that may be related or not to fractures. | Bell holes may be related to the corrosion in environments saturated by guano or to the runoff concentration in old vertical canaliculi, both in ferruginous caves. | H |
| Skylight | Collapse of ceiling parts intercepting the ground surface | Lift loss by gravitational action that may be related to geological structures like failures and fractures commonly observed in ferruginous caves where the rocky package is thin. | I |
| *Alveolus* | Small holes with milimetric to centimentric dimensions with circular to elliptical morphology. | They may present dissolutionary origin in low density zones that may be formed in the first speleogenesis phase in phreatic environments. | J |
| *Biotubule* | Rectilinear or curved tube-shaped relic features projected from conduit walls and ceilings with centimetric length and diameter. | Probable biological origin (by invertebrates or roots) sustained by thin layers of iron oxyhydroxide. | K |
| Paleoburrows | Grooves with centimetric dimensions excavated in the conduit walls and ceiling. | May be originated from claws of extinct animals. | L |
| Strutural overhang | Staggered rectangular or sub-rectangular points (dissymmetrical) projected from the ceiling. | Formed by the intersection of geological structures like bedrock and fracture. | M |
| Boxwork | Feature resultant from the rock preferential dissolution/erosion between the web of less soluble veins or mineralizations projected from the walls. | Formed by the differential erosion of embedding rock and veins. | N |
