# Supplementary material for: Extending beyond individual caves: a graph theory approach broadening conservation priorities in Amazon iron ore caves
Source: PeerJ. 2024 Jan 31;12:e16877. doi: 10.7717/peerj.16877 (PMC10838110; doi:10.7717/peerj.16877)
Supplement: Supplemental Information 5 [file peerj-12-16877-s005.docx]

| Species/Morphospecies | Genus | Family | Order | Class | Phylum |
| --- | --- | --- | --- | --- | --- |
| *Epiperipatus* sp.1 | *Epiperipatus* | Peripatidae | Euonychophora | Udeonychophora | Onychophora |
| *Flirtea valida* | *Flirtea* | Cosmetidae | Opiliones | Arachnida | Arthropoda |
| *Roquettea* sp.1 | *Roquettea* | Cosmetidae | Opiliones | Arachnida | Arthropoda |
| *Roquettea* sp.2 | *Roquettea* | Cosmetidae | Opiliones | Arachnida | Arthropoda |
| Escadabiidae sp.1 | gen.1 | Escadabiidae | Opiliones | Arachnida | Arthropoda |
| Gonyleptidae sp.1 | gen.1 | Gonyleptidae | Opiliones | Arachnida | Arthropoda |
| *Gerdesius* sp.1 | *Gerdesius* | Gerdesiidae | Opiliones | Arachnida | Arthropoda |
| *Saramacia annulata* | *Saramacia* | Manaosbiidae | Opiliones | Arachnida | Arthropoda |
| *Canga renatae* | *Canga* | Neogoveidae | Opiliones | Arachnida | Arthropoda |
| Gagrellinae sp.1 | gen.1 | Sclerosomatidae | Opiliones | Arachnida | Arthropoda |
| *Pickeliana* sp.1 | *Pickeliana* | Stygnidae | Opiliones | Arachnida | Arthropoda |
| *Protimesius* aff. *gracilis* | *Protimesius* | Stygnidae | Opiliones | Arachnida | Arthropoda |
| *Protimesius laevis* | *Protimesius* | Stygnidae | Opiliones | Arachnida | Arthropoda |
| *Zalmoxidae* sp.1 | gen.1 | Zalmoxidae | Opiliones | Arachnida | Arthropoda |
| *Ananteris luciae* | *Ananteris* | Buthidae | Scorpiones | Arachnida | Arthropoda |
| *Tityus bastosi* | *Tityus* | Buthidae | Scorpiones | Arachnida | Arthropoda |
| *Allokoenenia* sp.1 | *Allokoenenia* | Eukoeneniidae | Palpigradi | Arachnida | Arthropoda |
| *Allokoenenia* sp.2 | *Allokoenenia* | Eukoeneniidae | Palpigradi | Arachnida | Arthropoda |
| *Eukoenenia* sp.1 | *Eukoenenia* | Eukoeneniidae | Palpigradi | Arachnida | Arthropoda |
| *Eukoenenia* sp.2 | *Eukoenenia* | Eukoeneniidae | Palpigradi | Arachnida | Arthropoda |
| *Leptokoenenia* sp.2 | *Leptokoenenia* | Eukoeneniidae | Palpigradi | Arachnida | Arthropoda |
| *Cryptocellus canga* | *Cryptocellus* | Ricinoididae | Ricinulei | Arachnida | Arthropoda |
| *Caribeacarus brasiliensis* | *Caribeacarus* | Opilioacaridae | Opiliacariformes | Arachnida | Arthropoda |
| *Diplothyrus schubarti* | *Diplothyrus schubarti* | Neothyridae | Holothyrida | Arachnida | Arthropoda |
| *Argas (Ornithodoros)* sp.1 | *Argas* | Argasidae | Ixodida | Arachnida | Arthropoda |
| *Argas (Ornithodoros)* sp.2 | *Argas* | Argasidae | Ixodida | Arachnida | Arthropoda |
| *Argas (Ornithodoros)* sp.3 | *Argas* | Argasidae | Ixodida | Arachnida | Arthropoda |
| *Amblyomma cajennense* | *Amblyomma* | Ixodidae | Ixodida | Arachnida | Arthropoda |
| *Amblyomma goeldii* | *Amblyomma* | Ixodidae | Ixodida | Arachnida | Arthropoda |
| *Amblyomma* sp.1 | *Amblyomma* | Ixodidae | Ixodida | Arachnida | Arthropoda |
| *Amblyomma* sp.3 | *Amblyomma* | Ixodidae | Ixodida | Arachnida | Arthropoda |
| *Amblyomma* sp.4 | *Amblyomma* | Ixodidae | Ixodida | Arachnida | Arthropoda |
| *Amblyomma* sp.5 | *Amblyomma* | Ixodidae | Ixodida | Arachnida | Arthropoda |
| *Amblyomma* sp.6 | *Amblyomma* | Ixodidae | Ixodida | Arachnida | Arthropoda |
| *Pseudanapis* sp.1 | *Pseudanapis* | Anapidae | Araneae | Arachnida | Arthropoda |
| *Alpaida* aff. *negro* | *Alpaida* | Araneidae | Araneae | Arachnida | Arthropoda |
| *Alpaida* sp.2 | *Alpaida* | Araneidae | Araneae | Arachnida | Arthropoda |
| Barychelidae gen.1 sp.1 | gen.1 | Barychelidae | Araneae | Arachnida | Arthropoda |
| *Nops* sp.1 | *Nops* | Caponiidae | Araneae | Arachnida | Arthropoda |
| *Corinna* sp.1 | *Corinna* | Corinnidae | Araneae | Arachnida | Arthropoda |
| *Creugas* sp.1 | *Creugas* | Corinnidae | Araneae | Arachnida | Arthropoda |
| *Paradiestus* sp.1 | *Paradiestus* | Corinnidae | Araneae | Arachnida | Arthropoda |
| *Tupirinna* sp.1 | *Tupirinna* | Corinnidae | Araneae | Arachnida | Arthropoda |
| *Isoctenus* sp.1 | *Isoctenus* | Ctenidae | Araneae | Arachnida | Arthropoda |
| *Nothroctenus* sp.1 | *Nothroctenus* | Ctenidae | Araneae | Arachnida | Arthropoda |
| *Ctenizidae* gen.1 sp.1 | gen.1 | Ctenizidae | Araneae | Arachnida | Arthropoda |
| *Misionella carajas* | *Misionella* | Filistatidae | Araneae | Arachnida | Arthropoda |
| *Neohahnia* sp.1 | *Neohahnia* | Hahniidae | Araneae | Arachnida | Arthropoda |
| *Agyneta* sp.1 | *Agyneta* | Linyphiidae | Araneae | Arachnida | Arthropoda |
| *Ero* sp.1 | *Ero* | Mimetidae | Araneae | Arachnida | Arthropoda |
| *Ochyrocera* sp.1 | *Ochyrocera* | Ochyroceratidae | Araneae | Arachnida | Arthropoda |
| *Speocera* sp.1 | *Speocera* | Ochyroceratidae | Araneae | Arachnida | Arthropoda |
| *Theotima* aff. *minuta* | *Theotima* | Ochyroceratidae | Araneae | Arachnida | Arthropoda |
| *Gamasomorpha* sp.1 | *Gamasomorpha* | Oonopidae | Araneae | Arachnida | Arthropoda |
| *Neotrops* sp.1 | *Neotrops* | Oonopidae | Araneae | Arachnida | Arthropoda |
| *Neoxyphinus* sp.1 | *Neoxyphinus* | Oonopidae | Araneae | Arachnida | Arthropoda |
| Oonopidae gen.n.1 sp.1 | gen.1 | Oonopidae | Araneae | Arachnida | Arthropoda |
| *Stenoonops* sp.1 | *Stenoonops* | Oonopidae | Araneae | Arachnida | Arthropoda |
| *Stenoonops* sp.3 | *Stenoonops* | Oonopidae | Araneae | Arachnida | Arthropoda |
| *Otiothops* sp.1 | *Otiothops* | Palpimanidae | Araneae | Arachnida | Arthropoda |
| *Paratropis* sp.1 | *Paratropis* | Paratropididae | Araneae | Arachnida | Arthropoda |
| *Ibotyporanga* sp.1 | *Ibotyporanga* | Pholcidae | Araneae | Arachnida | Arthropoda |
| *Mesabolivar* sp.1 | *Mesabolivar* | Pholcidae | Araneae | Arachnida | Arthropoda |
| *Mesabolivar* sp.2 | *Mesabolivar* | Pholcidae | Araneae | Arachnida | Arthropoda |
| *Mesabolivar* sp.3 | *Mesabolivar* | Pholcidae | Araneae | Arachnida | Arthropoda |
| *Metagonia* sp.1 | *Metagonia* | Pholcidae | Araneae | Arachnida | Arthropoda |
| *Ninetinae* sp.1 | gen.1 | Pholcidae | Araneae | Arachnida | Arthropoda |
| aff. *Lygromma* sp.2 | gen.1 | Prodidomidae | Araneae | Arachnida | Arthropoda |
| *Lygromma* sp.1 | *Lygromma* | Prodidomidae | Araneae | Arachnida | Arthropoda |
| *Asaphobelis* sp.1 | *Asaphobelis* | Salticidae | Araneae | Arachnida | Arthropoda |
| *Soesilarischius* sp.1 | *Soesilarischius* | Salticidae | Araneae | Arachnida | Arthropoda |
| *Soesilarischius* sp.2 | *Soesilarischius* | Salticidae | Araneae | Arachnida | Arthropoda |
| *Soesilarischius* sp.3 | *Soesilarischius* | Salticidae | Araneae | Arachnida | Arthropoda |
| *Scytodes* sp.1 | *Scytodes* | Scytodidae | Araneae | Arachnida | Arthropoda |
| *Scytodes* sp.2 | *Scytodes* | Scytodidae | Araneae | Arachnida | Arthropoda |
| *Ariadna* sp.1 | *Ariadna* | Segestriidae | Araneae | Arachnida | Arthropoda |
| *Anapistula* sp.1 | *Anapistula* | Symphytognathidae | Araneae | Arachnida | Arthropoda |
| aff. *Matta* sp.1 | gen.1 | Tetrablemmidae | Araneae | Arachnida | Arthropoda |
| *Azilia histrio* | *Azilia* | Tetragnathidae | Araneae | Arachnida | Arthropoda |
| *Acanthoscurria theraphosoides* | *Acanthoscurria* | Theraphosidae | Araneae | Arachnida | Arthropoda |
| *Magulla* sp.1 | *Magulla* | Theraphosidae | Araneae | Arachnida | Arthropoda |
| *Stemmops* sp.1 | *Stemmops* | Theridiidae | Araneae | Arachnida | Arthropoda |
| *Theridion* sp.1 | *Theridion* | Theridiidae | Araneae | Arachnida | Arthropoda |
| *Theridion* sp.2 | *Theridion* | Theridiidae | Araneae | Arachnida | Arthropoda |
| *Thymoites* sp.1 | *Thymoites* | Theridiidae | Araneae | Arachnida | Arthropoda |
| *Plato* sp.1 | *Plato* | Theridiosomatidae | Araneae | Arachnida | Arthropoda |
| *Misumenops* sp.1 | *Misumenops* | Thomisidae | Araneae | Arachnida | Arthropoda |
| *Trechalea* sp.1 | *Trechalea* | Trechaleidae | Araneae | Arachnida | Arthropoda |
| *Uloborus* sp.1 | *Uloborus* | Uloboridae | Araneae | Arachnida | Arthropoda |
| *Charinus carajas* | *Charinus* | Charinidae | Amblypygi | Arachnida | Arthropoda |
| *Heterophrynus longicornis* | *Heterophrynus* | Phrynidae | Amblypygi | Arachnida | Arthropoda |
| *Cangazomus xikrin* | *Cangazomus* | Hubbardiidae | Schizomida | Arachnida | Arthropoda |
| *Cangazomus* sp.1 | *Cangazomus* | Hubbardiidae | Schizomida | Arachnida | Arthropoda |
| *Naderiore carajas* | *Naderiore* | Hubbardiidae | Schizomida | Arachnida | Arthropoda |
| Ballophilidae gen.1 sp.1 | gen.1 | Ballophilidae | Geophilomorpha | Chilopoda | Arthropoda |
| *Ityphilus* sp.1 | *Ityphilus* | Ballophilidae | Geophilomorpha | Chilopoda | Arthropoda |
| Geophilidae gen.1 sp.1 | *Geophilidae* | Geophilidae | Geophilomorpha | Chilopoda | Arthropoda |
| *Cryptops* sp.1 | *Cryptops* | Cryptopidae | Scolopendromorpha | Chilopoda | Arthropoda |
| *Otostigmus* aff. *amazonae* | *Otostigmus* | Scolopendridae | Scolopendromorpha | Chilopoda | Arthropoda |
| *Otostigmus* sp.1 | *Otostigmus* | Scolopendridae | Scolopendromorpha | Chilopoda | Arthropoda |
| *Scolopendropsis* sp.1 | *Scolopendropsis* | Scolopendridae | Scolopendromorpha | Chilopoda | Arthropoda |
| *Newportia (Newportia) ernsti fossulata* | *Newportia* | Scolopocryptopidae | Scolopendromorpha | Chilopoda | Arthropoda |
| *Newportia (Newportia)* sp.1 | *Newportia* | Scolopocryptopidae | Scolopendromorpha | Chilopoda | Arthropoda |
| *Newportia (Tidops) nisargani* | *Newportia* | Scolopocryptopidae | Scolopendromorpha | Chilopoda | Arthropoda |
| *Scolopocryptops miersii* | *Scolopocryptops* | Scolopocryptopidae | Scolopendromorpha | Chilopoda | Arthropoda |
| *Sphendononema guindingii* | *Sphendononema* | Pselliodidae | Scutigeromorpha | Chilopoda | Arthropoda |
| *Scolopendrellopsis* sp.1 | *Scolopendrellopsis* | Scolopendrellidae | Symphyla | Symphyla | Arthropoda |
| cf. *Scutigerella* sp.1 | *Scutigerella* | Scutigerellidae | Symphyla | Symphyla | Arthropoda |
| *Hanseniella* sp.1 | *Hanseniella* | Scutigerellidae | Symphyla | Symphyla | Arthropoda |
| Pauropodidae sp.1 | gen.1 | Pauropodidae | Pauropoda | Pauropoda | Arthropoda |
| Pauropodidae sp.2 | gen.1 | Pauropodidae | Pauropoda | Pauropoda | Arthropoda |
| Spirostreptidae gen.1 sp.1 | gen.1 | Spirostreptidae | Spirostreptida | Diplopoda | Arthropoda |
| *Glomeridesmus* sp.1 | *Glomeridesmus* | Glomeridesmidae | Glomeridesmida | Diplopoda | Arthropoda |
| *Parastenonia* sp.1 | *Parastenonia* | Chelodesmidae | Polydesmida | Diplopoda | Arthropoda |
| Fuhrmannodesmidae gen.1 sp.1 | gen.1 | Fuhrmannodesmidae | Polydesmida | Diplopoda | Arthropoda |
| Pyrgodesmidae gen.1 sp.2 | gen.1 | Pyrgodesmidae | Polydesmida | Diplopoda | Arthropoda |
| *Polyxenidae* sp.1 | gen.1 | Polyxenidae | Polyxenida | Diplopoda | Arthropoda |
| *Macrobrachium* sp.1 | *Macrobrachium* | Palaemonidae | Decapoda | Malacostraca | Arthropoda |
| *Microthelphusa somanni* | *Microthelphusa* | Pseudothelphusidae | Decapoda | Malacostraca | Arthropoda |
| cf. *Bogidiella* sp.1 | *Bogidiella* | Bogidiellidae | Amphipoda | Malacostraca | Arthropoda |
| *Hyalella* sp.2 | *Hyalella* | Hyalellidae | Amphipoda | Malacostraca | Arthropoda |
| *Ptenothrix* sp.1 | *Ptenothrix* | Dicyrtomidae | Symphypleona | Entognatha | Arthropoda |
| *Campylothorax* sp.4 | *Campylothorax* | Entomobryidae | Entomobryomorpha | Entognatha | Arthropoda |
| *Lepidonella* sp.7 | *Lepidonella* | Entomobryidae | Entomobryomorpha | Entognatha | Arthropoda |
| *Trogolaphysa* sp.2 | *Trogolaphysa* | Entomobryidae | Entomobryomorpha | Entognatha | Arthropoda |
| *Mastigoceras* sp.4 | *Mastigoceras* | Heteromuridae | Entomobryomorpha | Entognatha | Arthropoda |
| *Willemia* sp.1 | *Willemia* | Hypogastruridae | Poduromorpha | Entognatha | Arthropoda |
| *Hemisotoma thermophila* | *Hemisotoma* | Isotomidae | Entomobryomorpha | Entognatha | Arthropoda |
| *Isotomiella nummulifer* | *Isotomiella* | Isotomidae | Entomobryomorpha | Entognatha | Arthropoda |
| *Cyphoderus agnotus* | *Cyphoderus* | Lepidocyrtidae | Entomobryomorpha | Entognatha | Arthropoda |
| *Cyphoderus innominatus* | *Cyphoderus* | Lepidocyrtidae | Entomobryomorpha | Entognatha | Arthropoda |
| *Cyphoderus similis* | *Cyphoderus* | Lepidocyrtidae | Entomobryomorpha | Entognatha | Arthropoda |
| *Cyphoderus* sp.7 | *Cyphoderus* | Lepidocyrtidae | Entomobryomorpha | Entognatha | Arthropoda |
| *Lepidocyrtus* sp.1 | *Lepidocyrtus* | Lepidocyrtidae | Entomobryomorpha | Entognatha | Arthropoda |
| *Lepidocyrtus* sp.2 | *Lepidocyrtus* | Lepidocyrtidae | Entomobryomorpha | Entognatha | Arthropoda |
| *Pseudosinella* sp.1 | *Pseudosinella* | Lepidocyrtidae | Entomobryomorpha | Entognatha | Arthropoda |
| *Neotropiella* sp.2 | *Neotropiella* | Neanuridae | Poduromorpha | Entognatha | Arthropoda |
| *Neotropiella* sp.3 | *Neotropiella* | Neanuridae | Poduromorpha | Entognatha | Arthropoda |
| *Oncopodura* sp.1 | *Oncopodura* | Oncopoduridae | Entomobryomorpha | Entognatha | Arthropoda |
| *Seira* ca. *mirianae* | *Seira* | Seiridae | Entomobryomorpha | Entognatha | Arthropoda |
| *Seira mendoncae* | *Seira* | Seiridae | Entomobryomorpha | Entognatha | Arthropoda |
| *Seira prodiga* | *Seira* | Seiridae | Entomobryomorpha | Entognatha | Arthropoda |
| *Allacma* sp.1 | *Allacma* | Sminthuridae | Symphypleona | Entognatha | Arthropoda |
| *Songhaica* sp.1 | *Songhaica* | Sminthuridae | Symphypleona | Entognatha | Arthropoda |
| Campodeidae gen1. sp.1 | gen.1 | Campodeidae | Diplura | Entognatha | Arthropoda |
| Campodeidae gen1. sp.2 | gen.1 | Campodeidae | Diplura | Entognatha | Arthropoda |
| Campodeidae gen2. sp.1 | gen.2 | Campodeidae | Diplura | Entognatha | Arthropoda |
| Campodeidae gen2. sp.2 | gen.2 | Campodeidae | Diplura | Entognatha | Arthropoda |
| Campodeidae gen2. sp.3 | gen.2 | Campodeidae | Diplura | Entognatha | Arthropoda |
| Japygidae gen1. sp.2 | gen.1 | Japygidae | Diplura | Entognatha | Arthropoda |
| Parajapygidae gen1. sp.1 | gen.1 | Parajapygidae | Diplura | Entognatha | Arthropoda |
| Projapygidae sp.1 | gen.1 | Projapygidae | Diplura | Entognatha | Arthropoda |
| Projapygidae sp.2 | gen.1 | Projapygidae | Diplura | Entognatha | Arthropoda |
| *Neomachilellus* sp.1 | *Neomachilellus* | Meinertellidae | Archaeognatha | Insecta | Arthropoda |
| cf. *Ctenolepisma* sp.1 | *Ctenolepisma* | Lepismatidae | Zygentoma | Insecta | Arthropoda |
| Atelurinae gen.1 sp.1 | gen.1 | Nicoletiidae | Zygentoma | Insecta | Arthropoda |
| Atelurinae gen.1 sp.2 | gen.1 | Nicoletiidae | Zygentoma | Insecta | Arthropoda |
| Atelurinae gen.1 sp.3 | gen.1 | Nicoletiidae | Zygentoma | Insecta | Arthropoda |
| Nicoletiinae gen.1 sp.1 | gen.2 | Nicoletiidae | Zygentoma | Insecta | Arthropoda |
| Nicoletiinae gen.1 sp.2 | gen.2 | Nicoletiidae | Zygentoma | Insecta | Arthropoda |
| Nicoletiinae gen.1 sp.3 | gen.2 | Nicoletiidae | Zygentoma | Insecta | Arthropoda |
| Blaberinae gen.1 sp.1 | gen.1 | Blaberidae | Blattaria | Insecta | Arthropoda |
| Blattellinae gen.1 sp.1 | gen.1 | Ectobiidae | Blattaria | Insecta | Arthropoda |
| Blattellinae gen.2 sp.1 | gen.2 | Ectobiidae | Blattaria | Insecta | Arthropoda |
| Blattellinae gen.3 sp.1 | gen.3 | Ectobiidae | Blattaria | Insecta | Arthropoda |
| Blattellinae gen.4 sp.1 | gen.4 | Ectobiidae | Blattaria | Insecta | Arthropoda |
| Blattellinae gen.4 sp.2 | gen.4 | Ectobiidae | Blattaria | Insecta | Arthropoda |
| Blattellinae gen.4 sp.6 | gen.4 | Ectobiidae | Blattaria | Insecta | Arthropoda |
| Holocompsinae gen.1 sp.1 | gen.1 | Holocompsidae | Blattaria | Insecta | Arthropoda |
| Polyphagidae gen.1 sp.1 | gen.1 | Polyphagidae | Blattaria | Insecta | Arthropoda |
| Polyphagidae gen.1 sp.2 | gen.1 | Polyphagidae | Blattaria | Insecta | Arthropoda |
| *Euphyllodromia* sp.1 | *Euphyllodromia* | Pseudophyllodromiidae | Blattaria | Insecta | Arthropoda |
| *Coptotermes* sp.1 | *Coptotermes* | Rhinotermitidae | Blattaria | Insecta | Arthropoda |
| *Heterotermes* sp.1 | *Heterotermes* | Rhinotermitidae | Blattaria | Insecta | Arthropoda |
| *Agnathotermes* sp.1 | *Agnathotermes* | Termitidae | Blattaria | Insecta | Arthropoda |
| *Anoplotermes* sp.1 | *Anoplotermes* | Termitidae | Blattaria | Insecta | Arthropoda |
| *Aparatermes* sp.1 | *Aparatermes* | Termitidae | Blattaria | Insecta | Arthropoda |
| *Armitermes* sp.1 | *Armitermes* | Termitidae | Blattaria | Insecta | Arthropoda |
| *Armitermes* sp.2 | *Armitermes* | Termitidae | Blattaria | Insecta | Arthropoda |
| *Armitermes* sp.3 | *Armitermes* | Termitidae | Blattaria | Insecta | Arthropoda |
| *Atlantitermes* sp.1 | *Atlantitermes* | Termitidae | Blattaria | Insecta | Arthropoda |
| *Atlantitermes* sp.2 | *Atlantitermes* | Termitidae | Blattaria | Insecta | Arthropoda |
| *Convexitermes* sp.1 | *Convexitermes* | Termitidae | Blattaria | Insecta | Arthropoda |
| *Dihoplotermes* sp.1 | *Dihoplotermes* | Termitidae | Blattaria | Insecta | Arthropoda |
| *Embiratermes* sp.1 | *Embiratermes* | Termitidae | Blattaria | Insecta | Arthropoda |
| *Grigiotermes* sp.1 | *Grigiotermes* | Termitidae | Blattaria | Insecta | Arthropoda |
| *Labiotermes* sp.1 | *Labiotermes* | Termitidae | Blattaria | Insecta | Arthropoda |
| *Nasutitermes* sp.1 | *Nasutitermes* | Termitidae | Blattaria | Insecta | Arthropoda |
| *Nasutitermes* sp.2 | *Nasutitermes* | Termitidae | Blattaria | Insecta | Arthropoda |
| *Syntermes* sp.1 | *Syntermes* | Termitidae | Blattaria | Insecta | Arthropoda |
| *Termes* sp.1 | *Termes* | Termitidae | Blattaria | Insecta | Arthropoda |
| Amphientomidae gen2. sp.1 | gen.2 | Amphientomidae | Psocoptera | Insecta | Arthropoda |
| Epipsocidae gen1. sp.1 | gen.1 | Epipsocidae | Psocoptera | Insecta | Arthropoda |
| Epipsocidae gen1. sp.2 | gen.1 | Epipsocidae | Psocoptera | Insecta | Arthropoda |
| Lepidopsocidae sp.1 | gen.1 | Lepidopsocidae | Psocoptera | Insecta | Arthropoda |
| Liposcelididae gen1. sp.1 | gen.1 | Liposcelididae | Psocoptera | Insecta | Arthropoda |
| Psyllipsocidae gen1. sp.1 | gen.1 | Psyllipsocidae | Psocoptera | Insecta | Arthropoda |
| Psyllipsocidae gen1. sp.2 | gen.1 | Psyllipsocidae | Psocoptera | Insecta | Arthropoda |
| Psyllipsocidae gen1. sp.3 | gen.1 | Psyllipsocidae | Psocoptera | Insecta | Arthropoda |
| Psyllipsocidae gen1. sp.4 | gen.1 | Psyllipsocidae | Psocoptera | Insecta | Arthropoda |
| *Ptiloneura* sp.1 | *Ptiloneura* | Ptiloneuridae | Psocoptera | Insecta | Arthropoda |
| *Acromyrmex* sp.1 | *Acromyrmex* | Formicidae | Hymenoptera | Insecta | Arthropoda |
| *Acropyga* sp.1 | *Acropyga* | Formicidae | Hymenoptera | Insecta | Arthropoda |
| *Anochetus* sp.1 | *Anochetus* | Formicidae | Hymenoptera | Insecta | Arthropoda |
| *Apterostigma* sp.3 | *Apterostigma* | Formicidae | Hymenoptera | Insecta | Arthropoda |
| *Brachymyrmex* sp.1 | *Brachymyrmex* | Formicidae | Hymenoptera | Insecta | Arthropoda |
| *Camponotus* sp.2 | *Camponotus* | Formicidae | Hymenoptera | Insecta | Arthropoda |
| *Camponotus* sp.3 | *Camponotus* | Formicidae | Hymenoptera | Insecta | Arthropoda |
| *Camponotus* sp.4 | *Camponotus* | Formicidae | Hymenoptera | Insecta | Arthropoda |
| *Camponotus* sp.6 | *Camponotus* | Formicidae | Hymenoptera | Insecta | Arthropoda |
| *Camponotus* sp.7 | *Camponotus* | Formicidae | Hymenoptera | Insecta | Arthropoda |
| *Cardiocondyla* sp.1 | *Cardiocondyla* | Formicidae | Hymenoptera | Insecta | Arthropoda |
| *Cardiocondyla* sp.2 | *Cardiocondyla* | Formicidae | Hymenoptera | Insecta | Arthropoda |
| *Carebara* sp.1 | *Carebara* | Formicidae | Hymenoptera | Insecta | Arthropoda |
| *Carebara* sp.2 | *Carebara* | Formicidae | Hymenoptera | Insecta | Arthropoda |
| *Cephalotes clypeatus* | *Cephalotes* | Formicidae | Hymenoptera | Insecta | Arthropoda |
| *Cephalotes* sp.4 | *Cephalotes* | Formicidae | Hymenoptera | Insecta | Arthropoda |
| *Cephalotes umbraculatus* | *Cephalotes* | Formicidae | Hymenoptera | Insecta | Arthropoda |
| *Crematogaster* sp.1 | *Crematogaster* | Formicidae | Hymenoptera | Insecta | Arthropoda |
| *Crematogaster* sp.2 | *Crematogaster* | Formicidae | Hymenoptera | Insecta | Arthropoda |
| *Cyphomyrmex* sp.2 | *Cyphomyrmex* | Formicidae | Hymenoptera | Insecta | Arthropoda |
| *Dolichoderus* sp.1 | *Dolichoderus* | Formicidae | Hymenoptera | Insecta | Arthropoda |
| *Ectatomma* sp.2 | *Ectatomma* | Formicidae | Hymenoptera | Insecta | Arthropoda |
| Formicidae gen.1 sp.2 | gen.1 | Formicidae | Hymenoptera | Insecta | Arthropoda |
| *Gnamptogenys* sp.1 | *Gnamptogenys* | Formicidae | Hymenoptera | Insecta | Arthropoda |
| *Gnamptogenys* sp.2 | *Gnamptogenys* | Formicidae | Hymenoptera | Insecta | Arthropoda |
| *Gnamptogenys* sp.4 | *Gnamptogenys* | Formicidae | Hymenoptera | Insecta | Arthropoda |
| *Hypoponera* sp.1 | *Hypoponera* | Formicidae | Hymenoptera | Insecta | Arthropoda |
| *Hypoponera* sp.2 | *Hypoponera* | Formicidae | Hymenoptera | Insecta | Arthropoda |
| *Hypoponera* sp.3 | *Hypoponera* | Formicidae | Hymenoptera | Insecta | Arthropoda |
| *Labidus* sp.1 | *Labidus* | Formicidae | Hymenoptera | Insecta | Arthropoda |
| *Labidus* sp.2 | *Labidus* | Formicidae | Hymenoptera | Insecta | Arthropoda |
| *Linepithema* sp.1 | *Linepithema* | Formicidae | Hymenoptera | Insecta | Arthropoda |
| *Mayaponera* sp.1 | *Mayaponera* | Formicidae | Hymenoptera | Insecta | Arthropoda |
| *Mayaponera* sp.4 | *Mayaponera* | Formicidae | Hymenoptera | Insecta | Arthropoda |
| *Myrmicocrypta* sp.1 | *Myrmicocrypta* | Formicidae | Hymenoptera | Insecta | Arthropoda |
| *Neivamyrmex* sp.1 | *Neivamyrmex* | Formicidae | Hymenoptera | Insecta | Arthropoda |
| *Neoponera* sp.1 | *Neoponera* | Formicidae | Hymenoptera | Insecta | Arthropoda |
| *Ochetomyrmex neopolitus* | *Ochetomyrmex* | Formicidae | Hymenoptera | Insecta | Arthropoda |
| *Octostruma* sp.1 | *Octostruma* | Formicidae | Hymenoptera | Insecta | Arthropoda |
| *Odontomachus* sp.1 | *Odontomachus* | Formicidae | Hymenoptera | Insecta | Arthropoda |
| *Pachycondyla* sp.1 | *Pachycondyla* | Formicidae | Hymenoptera | Insecta | Arthropoda |
| *Pachycondyla* sp.2 | *Pachycondyla* | Formicidae | Hymenoptera | Insecta | Arthropoda |
| *Paratrechina* sp.1 | *Paratrechina* | Formicidae | Hymenoptera | Insecta | Arthropoda |
| *Paratrechina* sp.2 | *Paratrechina* | Formicidae | Hymenoptera | Insecta | Arthropoda |
| *Pheidole* sp.1 | *Pheidole* | Formicidae | Hymenoptera | Insecta | Arthropoda |
| *Pheidole* sp.11 | *Pheidole* | Formicidae | Hymenoptera | Insecta | Arthropoda |
| *Pheidole* sp.13 | *Pheidole* | Formicidae | Hymenoptera | Insecta | Arthropoda |
| *Pheidole* sp.2 | *Pheidole* | Formicidae | Hymenoptera | Insecta | Arthropoda |
| *Pheidole* sp.4 | *Pheidole* | Formicidae | Hymenoptera | Insecta | Arthropoda |
| *Pheidole* sp.6 | *Pheidole* | Formicidae | Hymenoptera | Insecta | Arthropoda |
| *Pheidole* sp.9 | *Pheidole* | Formicidae | Hymenoptera | Insecta | Arthropoda |
| *Ponerinae* sp.2 | *Ponerinae* | Formicidae | Hymenoptera | Insecta | Arthropoda |
| *Procryptocerus* sp.1 | *Procryptocerus* | Formicidae | Hymenoptera | Insecta | Arthropoda |
| *Rogeria* sp.2 | *Rogeria* | Formicidae | Hymenoptera | Insecta | Arthropoda |
| *Rogeria* sp.3 | *Rogeria* | Formicidae | Hymenoptera | Insecta | Arthropoda |
| *Rogeria* sp.4 | *Rogeria* | Formicidae | Hymenoptera | Insecta | Arthropoda |
| *Solenopsis* sp.2 | *Solenopsis* | Formicidae | Hymenoptera | Insecta | Arthropoda |
| *Solenopsis* sp.3 | *Solenopsis* | Formicidae | Hymenoptera | Insecta | Arthropoda |
| *Solenopsis* sp.5 | *Solenopsis* | Formicidae | Hymenoptera | Insecta | Arthropoda |
| *Solenopsis* sp.7 | *Solenopsis* | Formicidae | Hymenoptera | Insecta | Arthropoda |
| *Strumigenys* sp.1 | *Strumigenys* | Formicidae | Hymenoptera | Insecta | Arthropoda |
| *Wasmannia* sp.1 | *Wasmannia* | Formicidae | Hymenoptera | Insecta | Arthropoda |
| cf. *Pulex* sp.1 | *Pulex* | Pulicidae | Siphonaptera | Insecta | Arthropoda |
| *Hagenulopsis* sp.1 | *Hagenulopsis* | Leptophlebiidae | Ephemeroptera | Insecta | Arthropoda |
