# Supplementary material for: Extending beyond individual caves: a graph theory approach broadening conservation priorities in Amazon iron ore caves
Source: PeerJ. 2024 Jan 31;12:e16877. doi: 10.7717/peerj.16877 (PMC10838110; doi:10.7717/peerj.16877)
Supplement: Supplemental Information 6 — The variable’s names, types, units and concepts (description) are in Table S1 and codebook. [file peerj-12-16877-s006.docx]

| Cave | Landscape features | | | | | | | | | | | | | Cave features | | | | | | | | | | | | | | |
| --- | --- | --- | --- | --- | --- | --- | --- | --- | --- | --- | --- | --- | --- | --- | --- | --- | --- | --- | --- | --- | --- | --- | --- | --- | --- | --- | --- | --- |
|  | UTM_E | UTM_N | Alt | Esc | Gcomp | Vapr | Tmin | Tmax | Tavg | Srad | Prec | ForC | CngC | Hp | Dpt | Area | Vol | Plan | Gran | Smf | Litho | BatG | FzC | MaxT | MinT | MaxH | MinH | WI |
| ST_0001 | 599910 | 9301568 | 723 | 10 | Shoulder | 2.366 | 16.950 | 29.092 | 23.025 | 15119.083 | 157.917 | 8.890 | 10.690 | 53.680 | 6.260 | 295.860 | 451 | CC_Mx | Clay | 4 | Other | 4.330 | A | 23.35 | 22.75 | 93 | 87 | I |
| ST_0002 | 597508 | 9300729 | 747 | 11 | Shoulder | 2.388 | 17.100 | 29.267 | 23.183 | 15156.583 | 157.833 | 12.590 | 6.990 | 9.060 | 2.490 | 11.240 | 25 | SC_Rt | Clay | 3 | Other | 0.000 | A | 24.8 | 23.4 | 96 | 82 | I |
| ST_0003 | 598050 | 9300548 | 691 | 7 | Shoulder | 2.386 | 17.092 | 29.258 | 23.158 | 15138.333 | 158.000 | 11.090 | 8.490 | 75.720 | 9.330 | 193.120 | 235 | CC_Sp | Clay | 10 | B+C | 0.890 | A | 23.9 | 23.9 | 99 | 89 | I |
| ST_0004 | 598103 | 9300524 | 726 | 4 | Shoulder | 2.386 | 17.092 | 29.258 | 23.158 | 15138.333 | 158.000 | 10.950 | 8.630 | 12.280 | 2.790 | 40.060 | 54 | SC_Rc | Pebble | 5 | B+C | 0.000 | A | 23.4 | 21.7 | 91 | 84 | I |
| ST_0005 | 598795 | 9300254 | 756 | 1 | Shoulder | 2.355 | 16.892 | 29.000 | 22.942 | 15139.000 | 158.333 | 13.150 | 6.430 | 8.720 | 2.380 | 30.800 | 23 | CC_Ms | Boulder | 3 | CNG | 0.130 | A | 24.6 | 22.6 | 90 | 83 | I |
| ST_0006 | 598950 | 9300324 | 738 | 5 | Shoulder | 2.355 | 16.892 | 29.000 | 22.942 | 15139.000 | 158.333 | 8.210 | 11.370 | 6.490 | 0.770 | 11.550 | 9 | SC_Fn | Clay | 3 | CNG | 0.000 | A | 25.6 | 24.3 | 89 | 63 | I |
| ST_0007 | 599007 | 9300311 | 750 | 6 | Shoulder | 2.355 | 16.892 | 29.000 | 22.942 | 15139.000 | 158.333 | 7.480 | 12.100 | 4.950 | 3.310 | 11.620 | 12 | SC_Sc | Pebble | 4 | CNG | 0.000 | A | 25.1 | 23.8 | 88 | 65 | I |
| ST_0008 | 598497 | 9300779 | 724 | 8 | Shoulder | 2.386 | 17.092 | 29.258 | 23.158 | 15138.333 | 158.000 | 7.190 | 12.390 | 10.680 | 4.010 | 17.460 | 9 | SC_Fn | Pebble | 3 | CNG | 0.000 | A | 23.9 | 23.8 | 88 | 81 | I |
| ST_0009 | 598539 | 9300832 | 729 | 3 | Shoulder | 2.394 | 17.133 | 29.325 | 23.233 | 15071.917 | 158.083 | 8.820 | 10.760 | 18.450 | 0.980 | 67.590 | 102 | CC_Mx | Boulder | 4 | BIF | 0.000 | A | 24.1 | 23.1 | 95 | 77 | I |
| ST_0010 | 598544 | 9300855 | 738 | 4 | Shoulder | 2.394 | 17.133 | 29.325 | 23.233 | 15071.917 | 158.083 | 9.740 | 9.840 | 16.460 | 1.220 | 40.990 | 63 | CC_Sp | Pebble | 4 | BIF | 0.000 | P | 23.9 | 23.2 | 92 | 87 | I |
| ST_0011 | 598516 | 9300790 | 720 | 1 | Shoulder | 2.394 | 17.133 | 29.325 | 23.233 | 15071.917 | 158.083 | 7.360 | 12.220 | 51.570 | 5.630 | 119.860 | 150 | CC_Sp | Clay | 4 | BIF | 1.910 | P | 24.1 | 24.1 | 95 | 75 | P |
| ST_0012 | 598563 | 9300890 | 731 | 6 | Shoulder | 2.447 | 17.250 | 29.650 | 23.450 | 15145.083 | 159.333 | 10.750 | 8.830 | 10.440 | 2.530 | 45.430 | 78 | CC_Mx | Boulder | 3 | B+C | 0.120 | A | 23.3 | 23.3 | 91 | 83 | I |
| ST_0013 | 598803 | 9301653 | 716 | 7 | Shoulder | 2.348 | 16.800 | 28.958 | 22.867 | 15063.833 | 158.500 | 14.460 | 5.120 | 24.030 | 1.980 | 72.140 | 209 | CC_Mx | Pebble | 8 | B+C | 0.000 | A | 23.9 | 23.7 | 86 | 85 | I |
| ST_0014 | 598939 | 9301985 | 682 | 3 | Shoulder | 2.428 | 17.383 | 29.642 | 23.508 | 15151.083 | 157.167 | 14.420 | 5.160 | 4.820 | 0.610 | 5.760 | 3 | SC_Fn | Cobble | 2 | CNG | 0.030 | A | 25.3 | 24.6 | 90 | 74 | I |
| ST_0015 | 596909 | 9300125 | 698 | 15 | Shoulder | 2.388 | 17.100 | 29.267 | 23.183 | 15156.583 | 157.833 | 13.980 | 5.600 | 8.780 | 4.600 | 18.140 | 15 | SC_Rt | Clay | 2 | Other | 0.000 | A | 24.6 | 24.3 | 96 | 63 | I |
| ST_0016 | 596673 | 9300281 | 740 | 4 | Summit | 2.347 | 16.792 | 28.900 | 22.842 | 15145.667 | 158.250 | 6.160 | 13.420 | 14.560 | 1.130 | 51.310 | 48 | CC_Ms | Clay | 4 | B+C | 0.080 | A | 24.8 | 23.6 | 96 | 71 | I |
| ST_0017 | 598902 | 9301876 | 688 | 5 | Shoulder | 2.428 | 17.383 | 29.642 | 23.508 | 15151.083 | 157.167 | 12.760 | 6.820 | 65.570 | 4.140 | 295.800 | 717 | CC_Sp | Boulder | 8 | B+C | 6.910 | A | 25.4 | 24.8 | 94 | 90 | P |
| ST_0018 | 597050 | 9300190 | 714 | 8 | Shoulder | 2.388 | 17.100 | 29.267 | 23.183 | 15156.583 | 157.833 | 14.870 | 4.710 | 25.480 | 6.590 | 49.830 | 53 | SC_Rt | Pebble | 2 | BIF | 0.000 | A | 23.4 | 21.1 | 95 | 75 | I |
| ST_0019 | 597066 | 9300712 | 762 | 2 | Shoulder | 2.388 | 17.100 | 29.267 | 23.183 | 15156.583 | 157.833 | 13.340 | 6.240 | 5.750 | 0.720 | 78.810 | 89 | SC_Sc | Boulder | 1 | CNG | 0.000 | A | 25.3 | 23 | 87 | 63 | I |
| ST_0020 | 597020 | 9300682 | 761 | 4 | Shoulder | 2.388 | 17.100 | 29.267 | 23.183 | 15156.583 | 157.833 | 13.000 | 7.000 | 5.910 | 0.980 | 12.420 | 12 | SC_Cv | Pebble | 1 | Other | 0.000 | A | 23.5 | 22.9 | 90 | 62 | I |
| ST_0021 | 596015 | 9301321 | 731 | 4 | Shoulder | 2.366 | 16.892 | 29.075 | 23.000 | 15085.917 | 158.000 | 17.790 | 1.790 | 24.140 | 2.350 | 52.200 | 66 | CC_Ms | Boulder | 5 | B+C | 0.000 | A | 25.2 | 23.3 | 86 | 64 | I |
| ST_0022 | 596029 | 9301401 | 714 | 6 | Footslope | 2.366 | 16.892 | 29.075 | 23.000 | 15085.917 | 158.000 | 17.730 | 1.850 | 9.950 | 0.710 | 16.280 | 18 | SC_Fn | Boulder | 2 | BIF | 0.000 | A | 23.7 | 23.6 | 90 | 77 | I |
| ST_0023 | 596263 | 9301471 | 715 | 4 | Footslope | 2.366 | 16.892 | 29.075 | 23.000 | 15085.917 | 158.000 | 18.270 | 1.310 | 32.060 | 3.810 | 78.210 | 106 | SC_Rt | Clay | 5 | B+C | 0.000 | A | 23.8 | 23.3 | 97 | 84 | I |
| ST_0024 | 596346 | 9301454 | 738 | 3 | Footslope | 2.366 | 16.892 | 29.075 | 23.000 | 15085.917 | 158.000 | 17.180 | 2.400 | 6.200 | 3.160 | 8.340 | 15 | SC_Rt | Cobble | 3 | BIF | 0.000 | A | 26.1 | 23.4 | 99 | 69 | I |
| ST_0025 | 596347 | 9301450 | 738 | 2 | Footslope | 2.366 | 16.892 | 29.075 | 23.000 | 15085.917 | 158.000 | 17.160 | 2.420 | 4.880 | 1.090 | 11.190 | 9 | SC_Fn | Pebble | 3 | BIF | 0.000 | A | 26.1 | 23.9 | 96 | 75 | I |
| ST_0026 | 597298 | 9300701 | 768 | 3 | Shoulder | 2.388 | 17.100 | 29.267 | 23.183 | 15156.583 | 157.833 | 13.030 | 6.550 | 21.670 | 4.550 | 41.680 | 21 | CC_Ms | Clay | 4 | CNG | 0.000 | A | 23.8 | 23.6 | 94 | 79 | I |
| ST_0027 | 597073 | 9301631 | 675 | 7 | Shoulder | 2.365 | 16.900 | 29.075 | 23.000 | 15084.000 | 158.083 | 16.150 | 3.430 | 13.650 | 6.380 | 64.000 | 90 | CC_Mx | Cobble | 2 | BIF | 0.000 | A | 25 | 23.8 | 95 | 81 | I |
| ST_0028 | 597071 | 9301624 | 662 | 4 | Shoulder | 2.365 | 16.900 | 29.075 | 23.000 | 15084.000 | 158.083 | 16.090 | 3.490 | 8.620 | 2.080 | 25.730 | 34 | SC_Cv | Pebble | 3 | BIF | 0.000 | A | 24 | 23.8 | 93 | 64 | I |
| ST_0029 | 597088 | 9301662 | 666 | 6 | Shoulder | 2.365 | 16.900 | 29.075 | 23.000 | 15084.000 | 158.083 | 15.960 | 3.620 | 7.040 | 1.240 | 41.930 | 84 | SC_Sc | Pebble | 5 | BIF | 0.000 | A | 24.1 | 24.1 | 92 | 66 | I |
| ST_0030 | 597285 | 9301760 | 636 | 4 | Footslope | 2.518 | 17.992 | 30.333 | 24.167 | 15115.667 | 156.500 | 13.860 | 5.720 | 73.720 | 19.570 | 661.830 | 871 | CC_Ms | Boulder | 5 | B+C | 2.830 | A | 23.8 | 22.9 | 97 | 86 | I |
| ST_0031 | 599604 | 9302376 | 674 | 1 | Shoulder | 2.441 | 17.492 | 29.750 | 23.617 | 15187.667 | 156.667 | 12.660 | 6.920 | 11.560 | 7.810 | 49.000 | 114 | CC_Ms | Boulder | 1 | Other | 0.000 | A | 24.2 | 22.4 | 95 | 81 | I |
| ST_0032 | 597434 | 9301713 | 662 | 2 | Shoulder | 2.518 | 17.992 | 30.333 | 24.167 | 15115.667 | 156.500 | 13.930 | 5.650 | 20.660 | 6.520 | 67.940 | 61 | SC_Ms | Boulder | 2 | B+C | 0.000 | P | 26.4 | 24.4 | 96 | 75 | I |
| ST_0033 | 597738 | 9300802 | 754 | 2 | Shoulder | 2.394 | 17.133 | 29.325 | 23.233 | 15071.917 | 158.083 | 13.200 | 6.400 | 20.390 | 1.860 | 46.890 | 48 | SC_Bf | Clay | 3 | BIF | 0.000 | A | 23.4 | 23.1 | 93 | 88 | I |
| ST_0034 | 598125 | 9301324 | 642 | 5 | Footslope | 2.394 | 17.133 | 29.325 | 23.233 | 15071.917 | 158.083 | 19.210 | 0.370 | 43.000 | 7.670 | 173.050 | 209 | SC_Rt | Clay | 3 | BIF | 0.710 | P | 22.9 | 22.6 | 93 | 92 | I |
| ST_0035 | 599337 | 9302067 | 675 | 15 | Shoulder | 2.428 | 17.383 | 29.642 | 23.508 | 15151.083 | 157.167 | 10.850 | 8.730 | 30.760 | 4.840 | 52.380 | 77 | SC_Fn | Clay | 3 | BIF | 0.090 | A | 23.6 | 22.1 | 95 | 79 | I |
| ST_0037 | 599604 | 9302364 | 669 | 2 | Shoulder | 2.441 | 17.492 | 29.750 | 23.617 | 15187.667 | 156.667 | 12.270 | 7.310 | 23.720 | 2.230 | 59.830 | 53 | SC_Cv | Boulder | 4 | CNG | 0.780 | P | 27.1 | 25.6 | 92 | 88 | P |
| ST_0038 | 599831 | 9302093 | 685 | 3 | Shoulder | 2.441 | 17.492 | 29.750 | 23.617 | 15187.667 | 156.667 | 12.820 | 6.760 | 15.360 | 1.930 | 50.420 | 74 | CC_Ms | Pebble | 4 | CNG | 0.000 | A | 24.7 | 23.8 | 91 | 73 | I |
| ST_0039A | 599659 | 9302336 | 687 | 7 | Shoulder | 2.441 | 17.492 | 29.750 | 23.617 | 15187.667 | 156.667 | 12.060 | 7.520 | 28.790 | 2.350 | 191.390 | 427 | SC_Sc | Clay | 4 | Other | 0.000 | P | 24.2 | 23.9 | 93 | 88 | I |
| ST_0039B | 599659 | 9302336 | 687 | 7 | Shoulder | 2.441 | 17.492 | 29.750 | 23.617 | 15187.667 | 156.667 | 12.060 | 7.520 | 31.090 | 6.410 | 166.720 | 303 | CC_Ms | Boulder | 5 | BIF | 0.000 | A | 25.1 | 24.1 | 91 | 83 | I |
| ST_0041 | 600319 | 9302067 | 513 | 5 | Footslope | 2.441 | 17.492 | 29.750 | 23.617 | 15187.667 | 156.667 | 19.440 | 0.140 | 79.040 | 8.100 | 536.710 | 1020 | SC_Sc | Boulder | 3 | Other | 4.110 | A | 25.4 | 25.4 | 99 | 89 | P |
| ST_0042 | 600329 | 9302073 | 501 | 10 | Footslope | 2.441 | 17.492 | 29.750 | 23.617 | 15187.667 | 156.667 | 19.440 | 0.140 | 117.060 | 4.880 | 1168.480 | 3339 | CC_Sp | Boulder | 7 | Other | 158.040 | P | 25.7 | 24.9 | 97 | 87 | P |
| ST_0043 | 600351 | 9302090 | 525 | 7 | Footslope | 2.441 | 17.492 | 29.750 | 23.617 | 15187.667 | 156.667 | 19.440 | 0.140 | 17.690 | 4.210 | 56.040 | 51 | CC_Mx | Clay | 4 | Other | 0.000 | P | 24.2 | 24 | 94 | 90 | I |
| ST_0044 | 596588 | 9301569 | 709 | 8 | Footslope | 2.366 | 16.892 | 29.075 | 23.000 | 15085.917 | 158.000 | 17.990 | 1.590 | 103.840 | 5.640 | 382.360 | 575 | CC_Sp | Boulder | 3 | B+C | 75.880 | P | 25.3 | 25 | 94 | 83 | P |
| ST_0045 | 596627 | 9301549 | 694 | 5 | Footslope | 2.366 | 16.892 | 29.075 | 23.000 | 15085.917 | 158.000 | 18.030 | 1.550 | 8.020 | 2.050 | 40.410 | 90 | SC_Rc | Boulder | 2 | BIF | 0.000 | A | 23.6 | 23.2 | 90 | 77 | I |
| ST_0046 | 596634 | 9301538 | 698 | 6 | Footslope | 2.366 | 16.892 | 29.075 | 23.000 | 15085.917 | 158.000 | 18.010 | 1.570 | 11.180 | 1.790 | 17.990 | 10 | SC_Rt | Boulder | 1 | BIF | 0.000 | A | 24.6 | 23.7 | 91 | 88 | P |
| ST_0047 | 596635 | 9301533 | 703 | 6 | Footslope | 2.366 | 16.892 | 29.075 | 23.000 | 15085.917 | 158.000 | 17.990 | 1.590 | 5.590 | 2.410 | 2.820 | 3 | SC_Rt | Pebble | 2 | B+C | 0.000 | A | 23.6 | 22.9 | 92 | 71 | I |
| ST_0048 | 596484 | 9301539 | 690 | 6 | Footslope | 2.366 | 16.892 | 29.075 | 23.000 | 15085.917 | 158.000 | 16.910 | 2.670 | 17.290 | 1.480 | 42.280 | 45 | SC_Fn | Pebble | 3 | BIF | 0.000 | A | 23.4 | 22.1 | 94 | 91 | I |
| ST_0049 | 596503 | 9301378 | 749 | 2 | Footslope | 2.366 | 16.892 | 29.075 | 23.000 | 15085.917 | 158.000 | 16.420 | 3.160 | 12.440 | 1.860 | 16.400 | 30 | SC_Fn | Clay | 3 | BIF | 0.000 | A | 25.9 | 23.8 | 91 | 64 | I |
| ST_0050 | 596396 | 9301626 | 636 | 2 | Footslope | 2.366 | 16.892 | 29.075 | 23.000 | 15085.917 | 158.000 | 18.310 | 1.270 | 13.270 | 3.910 | 17.410 | 17 | SC_Ms | Pebble | 5 | BIF | 0.000 | A | 24 | 24 | 94 | 86 | I |
| ST_0051 | 595619 | 9301339 | 656 | 8 | Footslope | 2.407 | 17.192 | 29.392 | 23.308 | 15093.583 | 157.917 | 19.580 | 0.000 | 7.860 | 0.510 | 23.120 | 29 | SC_Rc | Pebble | 5 | BIF | 0.070 | A | 26.7 | 23.6 | 91 | 60 | I |
| ST_0052 | 595203 | 9301156 | 708 | 12 | Shoulder | 2.407 | 17.192 | 29.392 | 23.308 | 15093.583 | 157.917 | 16.240 | 3.340 | 9.430 | 2.170 | 54.850 | 124 | SC_Fn | Boulder | 4 | B+C | 0.000 | A | 24 | 23.5 | 94 | 90 | I |
| ST_0053 | 595205 | 9301186 | 703 | 7 | Shoulder | 2.407 | 17.192 | 29.392 | 23.308 | 15093.583 | 157.917 | 16.770 | 2.810 | 10.060 | 5.780 | 21.980 | 22 | SC_Fn | Boulder | 3 | CNG | 0.000 | A | 25.2 | 24.1 | 90 | 84 | I |
| ST_0054 | 595209 | 9301192 | 704 | 7 | Shoulder | 2.407 | 17.192 | 29.392 | 23.308 | 15093.583 | 157.917 | 16.950 | 2.630 | 65.300 | 8.840 | 211.790 | 204 | CC_Sp | Pebble | 5 | B+C | 3.220 | A | 25.5 | 24.4 | 97 | 94 | I |
| ST_0055 | 595604 | 9301303 | 681 | 2 | Footslope | 2.407 | 17.192 | 29.392 | 23.308 | 15093.583 | 157.917 | 19.580 | 0.000 | 11.560 | 2.470 | 16.680 | 11 | SC_Rt | Pebble | 1 | BIF | 0.000 | A | 23.5 | 22.6 | 90 | 82 | I |
| ST_0056 | 595125 | 9300495 | 772 | 0 | Summit | 2.344 | 16.767 | 28.875 | 22.825 | 15155.583 | 158.167 | 10.680 | 8.900 | 62.520 | 10.230 | 440.190 | 1933 | CC_Ms | Boulder | 7 | Other | 87.040 | P | 22.6 | 21.9 | 99 | 90 | P |
| ST_0057 | 595051 | 9301177 | 701 | 4 | Shoulder | 2.407 | 17.192 | 29.392 | 23.308 | 15093.583 | 157.917 | 14.010 | 5.570 | 8.870 | 1.380 | 21.420 | 21 | SC_Rt | Cobble | 1 | B+C | 0.000 | A | 24.1 | 24.1 | 99 | 57 | I |
| ST_0058 | 594912 | 9301120 | 704 | 4 | Shoulder | 2.442 | 17.467 | 29.692 | 23.567 | 15105.750 | 157.750 | 12.950 | 6.630 | 10.690 | 3.620 | 29.600 | 25 | CC_Mx | Pebble | 1 | BIF | 0.000 | A | 24.8 | 23.9 | 99 | 74 | I |
| ST_0059 | 594872 | 9301061 | 708 | 10 | Shoulder | 2.442 | 17.467 | 29.692 | 23.567 | 15105.750 | 157.750 | 11.650 | 7.930 | 6.300 | 1.300 | 5.370 | 4 | SC_Rt | Pebble | 6 | B+C | 0.000 | A | 25.1 | 24.2 | 99 | 76 | I |
| ST_0060 | 594879 | 9301059 | 710 | 10 | Shoulder | 2.442 | 17.467 | 29.692 | 23.567 | 15105.750 | 157.750 | 11.680 | 7.900 | 11.170 | 1.480 | 12.130 | 7 | SC_Bf | Pebble | 5 | B+C | 0.000 | A | 25.8 | 24.7 | 99 | 66 | I |
| ST_0061 | 594904 | 9301138 | 694 | 8 | Shoulder | 2.442 | 17.467 | 29.692 | 23.567 | 15105.750 | 157.750 | 13.540 | 6.040 | 24.300 | 1.940 | 53.220 | 58 | CC_Ms | Pebble | 4 | BIF | 0.250 | A | 24.3 | 24.3 | 99 | 83 | I |
| ST_0062 | 594811 | 9300985 | 716 | 15 | Shoulder | 2.442 | 17.467 | 29.692 | 23.567 | 15105.750 | 157.750 | 10.470 | 9.110 | 10.850 | 2.050 | 26.780 | 36 | SC_Rc | Boulder | 3 | CNG | 0.000 | A | 23.8 | 23.3 | 94 | 64 | I |
| ST_0063 | 594729 | 9301167 | 674 | 11 | Shoulder | 2.442 | 17.467 | 29.692 | 23.567 | 15105.750 | 157.750 | 15.160 | 4.420 | 15.410 | 8.770 | 102.740 | 440 | SC_Sc | Cobble | 3 | BIF | 0.000 | A | 25.5 | 23.6 | 89 | 59 | I |
| ST_0064 | 594760 | 9301119 | 704 | 8 | Shoulder | 2.442 | 17.467 | 29.692 | 23.567 | 15105.750 | 157.750 | 12.890 | 6.690 | 16.720 | 4.560 | 49.970 | 77 | SC_Fn | Boulder | 4 | B+C | 0.000 | A | 23.8 | 23.6 | 94 | 65 | I |
| ST_0065 | 594758 | 9301100 | 705 | 8 | Shoulder | 2.442 | 17.467 | 29.692 | 23.567 | 15105.750 | 157.750 | 12.320 | 7.260 | 6.080 | 1.350 | 7.790 | 4 | SC_Fn | Boulder | 2 | B+C | 0.000 | A | 25.6 | 23.6 | 93 | 63 | I |
| ST_0066 | 594335 | 9301108 | 691 | 4 | Shoulder | 2.442 | 17.467 | 29.692 | 23.567 | 15105.750 | 157.750 | 12.680 | 6.900 | 9.850 | 1.790 | 22.900 | 17 | SC_Fn | Clay | 1 | CNG | 0.130 | A | 25.5 | 25 | 94 | 68 | I |
| ST_0067 | 594431 | 9301118 | 634 | 5 | Footslope | 2.442 | 17.467 | 29.692 | 23.567 | 15105.750 | 157.750 | 13.580 | 6.000 | 6.540 | 1.590 | 8.940 | 13 | SC_Fn | Boulder | 1 | BIF | 0.000 | A | 25.3 | 23.5 | 95 | 69 | I |
| ST_0068 | 596257 | 9301488 | 702 | 4 | Footslope | 2.366 | 16.892 | 29.075 | 23.000 | 15085.917 | 158.000 | 18.490 | 1.090 | 6.800 | 0.780 | 8.640 | 8 | SC_Bf | Clay | 3 | BIF | 0.000 | A | 25.1 | 23.4 | 86 | 81 | I |
| ST_0069 | 596263 | 9301464 | 709 | 4 | Footslope | 2.366 | 16.892 | 29.075 | 23.000 | 15085.917 | 158.000 | 18.190 | 1.390 | 15.860 | 5.610 | 28.650 | 23 | SC_Ms | Boulder | 2 | BIF | 0.000 | A | 24.7 | 23.3 | 95 | 68 | I |
| ST_0070 | 597190 | 9301691 | 671 | 5 | Shoulder | 2.365 | 16.900 | 29.075 | 23.000 | 15084.000 | 158.083 | 13.570 | 6.010 | 5.580 | 1.130 | 21.450 | 39 | SC_Fn | Pebble | 4 | BIF | 0.000 | A | 24.6 | 23.7 | 88 | 66 | I |
