# Supplementary material for: Extending beyond individual caves: a graph theory approach broadening conservation priorities in Amazon iron ore caves
Source: PeerJ. 2024 Jan 31;12:e16877. doi: 10.7717/peerj.16877 (PMC10838110; doi:10.7717/peerj.16877)
Supplement: Supplemental Information 7 — For each cave, information is provided on the number of troglobitic species, connections established to other cavities (Degree), the uniqueness in relation to its troglobitic fauna and habitat specificity (Singularity), and the intermediation centrality measure (Betweenness), which indicates the potential of the cave as “bridges” between distant regions of the graph. [file peerj-12-16877-s007.docx]

| Caves | Number of troglobites | Degree | Singularity | Betwenness |  | Caves | Number of troglobites | Degree | Singularity | Betwenness |
| --- | --- | --- | --- | --- | --- | --- | --- | --- | --- | --- |
| ST_0001 | 3 | 4 | 5.667 | 0.000 |  | ST_0037 | 1 | 5 | 1.511 | 0.000 |
| ST_0002 | 1 | 9 | 0.840 | 7.451 |  | ST_0038 | 1 | 17 | 0.444 | 219.027 |
| ST_0003 | 3 | 9 | 2.519 | 8.746 |  | ST_0039A | 3 | 8 | 2.833 | 6.129 |
| ST_0004 | 1 | 21 | 0.360 | 87.165 |  | ST_0039B | 1 | 13 | 0.581 | 149.181 |
| ST_0005 | 1 | 10 | 0.756 | 7.628 |  | ST_0041 | 5 | 6 | 6.296 | 1.809 |
| ST_0006 | 0 | 9 | 0.000 | 11.932 |  | ST_0042 | 5 | 6 | 6.296 | 1.809 |
| ST_0007 | 1 | 13 | 0.581 | 16.797 |  | ST_0043 | 2 | 6 | 2.519 | 1.809 |
| ST_0008 | 0 | 14 | 0.000 | 46.366 |  | ST_0044 | 2 | 18 | 0.840 | 5.657 |
| ST_0009 | 1 | 14 | 0.540 | 45.400 |  | ST_0045 | 1 | 23 | 0.329 | 67.843 |
| ST_0010 | 1 | 21 | 0.360 | 88.772 |  | ST_0046 | 1 | 18 | 0.420 | 5.657 |
| ST_0011 | 2 | 10 | 1.511 | 1.758 |  | ST_0047 | 1 | 25 | 0.302 | 36.153 |
| ST_0012 | 1 | 17 | 0.444 | 103.735 |  | ST_0048 | 1 | 23 | 0.329 | 22.298 |
| ST_0013 | 1 | 7 | 1.079 | 11.726 |  | ST_0049 | 0 | 21 | 0.000 | 60.991 |
| ST_0014 | 1 | 21 | 0.360 | 126.414 |  | ST_0050 | 1 | 22 | 0.343 | 36.162 |
| ST_0015 | 0 | 7 | 0.000 | 13.288 |  | ST_0051 | 1 | 22 | 0.343 | 81.640 |
| ST_0016 | 2 | 9 | 1.679 | 6.197 |  | ST_0052 | 1 | 17 | 0.444 | 19.415 |
| ST_0017 | 3 | 3 | 7.556 | 0.000 |  | ST_0053 | 1 | 21 | 0.360 | 133.832 |
| ST_0018 | 1 | 11 | 0.687 | 15.950 |  | ST_0054 | 3 | 14 | 1.619 | 7.957 |
| ST_0019 | 0 | 11 | 0.000 | 26.953 |  | ST_0055 | 0 | 23 | 0.000 | 146.476 |
| ST_0020 | 1 | 20 | 0.378 | 105.135 |  | ST_0056 | 2 | 11 | 1.374 | 0.000 |
| ST_0021 | 2 | 17 | 0.889 | 17.033 |  | ST_0057 | 0 | 18 | 0.000 | 24.339 |
| ST_0022 | 1 | 22 | 0.343 | 63.340 |  | ST_0058 | 1 | 18 | 0.420 | 24.339 |
| ST_0023 | 2 | 18 | 0.840 | 32.646 |  | ST_0059 | 0 | 14 | 0.000 | 2.152 |
| ST_0024 | 1 | 18 | 0.420 | 14.812 |  | ST_0060 | 0 | 14 | 0.000 | 2.152 |
| ST_0025 | 0 | 21 | 0.000 | 24.254 |  | ST_0061 | 3 | 16 | 1.417 | 11.471 |
| ST_0026 | 1 | 10 | 0.756 | 26.011 |  | ST_0062 | 1 | 14 | 0.540 | 2.152 |
| ST_0027 | 1 | 22 | 0.343 | 78.800 |  | ST_0063 | 0 | 15 | 0.000 | 24.529 |
| ST_0028 | 1 | 20 | 0.378 | 39.074 |  | ST_0064 | 0 | 16 | 0.000 | 25.941 |
| ST_0029 | 1 | 19 | 0.398 | 25.373 |  | ST_0065 | 0 | 17 | 0.000 | 31.234 |
| ST_0030 | 4 | 9 | 3.358 | 1.174 |  | ST_0066 | 0 | 14 | 0.000 | 69.716 |
| ST_0031 | 2 | 9 | 1.679 | 33.148 |  | ST_0067 | 1 | 16 | 0.472 | 47.944 |
| ST_0032 | 3 | 7 | 3.238 | 10.446 |  | ST_0068 | 0 | 16 | 0.000 | 8.986 |
| ST_0033 | 1 | 10 | 0.756 | 29.415 |  | ST_0069 | 1 | 21 | 0.360 | 60.698 |
| ST_0034 | 3 | 7 | 3.238 | 2.732 |  | ST_0070 | 1 | 20 | 0.378 | 62.407 |
| ST_0035 | 2 | 9 | 1.679 | 30.424 |  |  |  |  |  |  |
