# Supplementary figures and images for: Extending beyond individual caves: a graph theory approach broadening conservation priorities in Amazon iron ore caves
Source: PeerJ. 2024 Jan 31;12:e16877. doi: 10.7717/peerj.16877 (PMC10838110; doi:10.7717/peerj.16877)

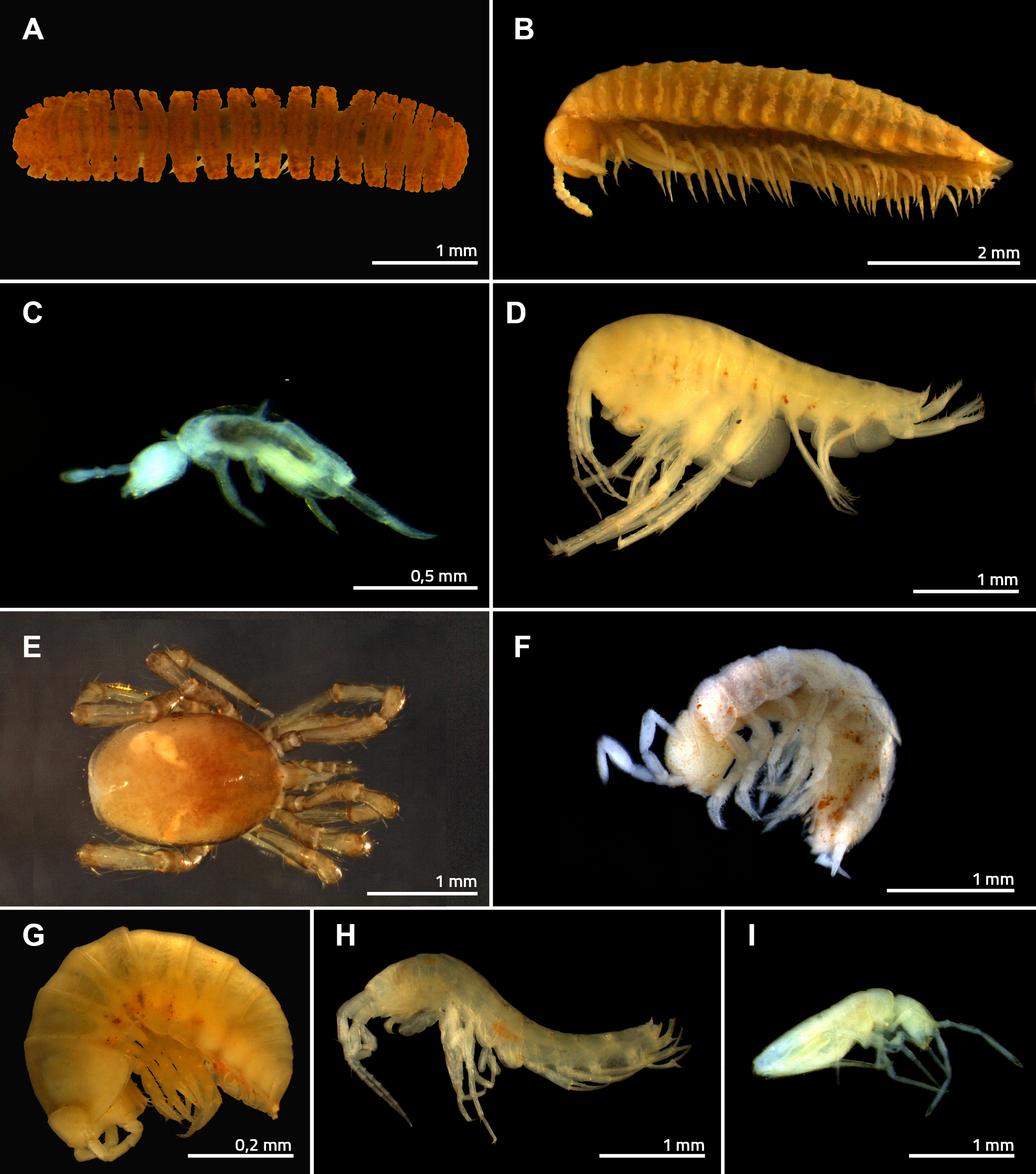

Supplement: Supplemental Information 8 — A) Pyrgodesmidae gen.1 sp.2 (Polydesmida: Pyrgodesmidae). B) Glomeridesmus sp.1 (Glomeridesmida: Glomeridesmidae). C) Pseudosinella sp.1 (Collembola: Lepidocyrtidae). D) Hyalella sp.2 (Amphipoda: Hyalellidae). E) Laelapidae sp.1 (Mesostigmata: Laelapidae). F) Trichorhina sp.1 (Isopoda: Platyarthridae). G) Circoniscus carajasensis (Isopoda: Scleropactidae). H) cf. Bogidiella sp.1 (Amphipoda: Bogidiellidae). I) Trogolaphysa sp.2 (Collembola: Entomobryidae). [file peerj-12-16877-s008.png]

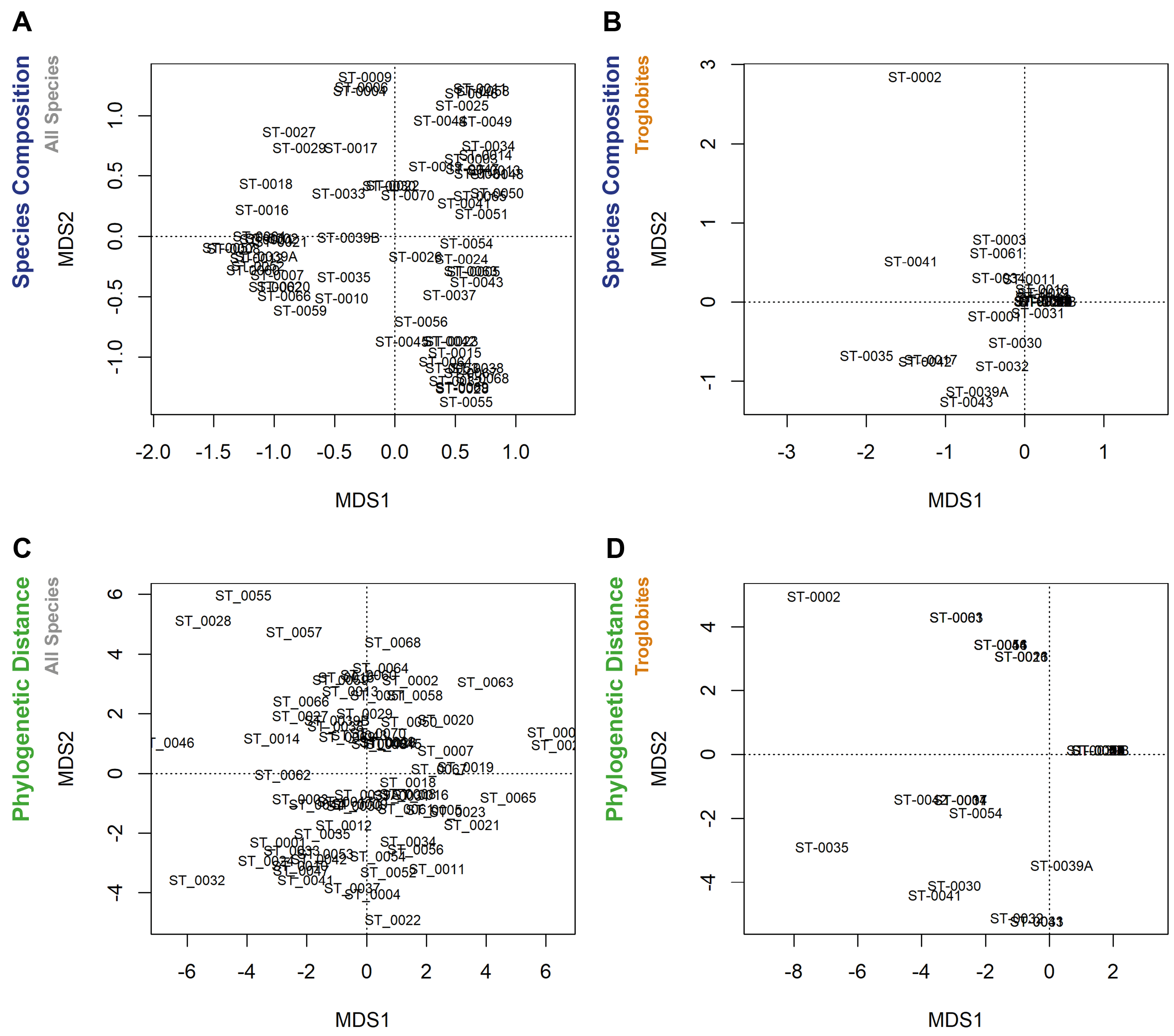

Supplement: Supplemental Information 9 — We carried out PCoA based on the Bray-Curtis Index for species composition and Euclidean distance for phylogenetic relationships. [file peerj-12-16877-s009.png]

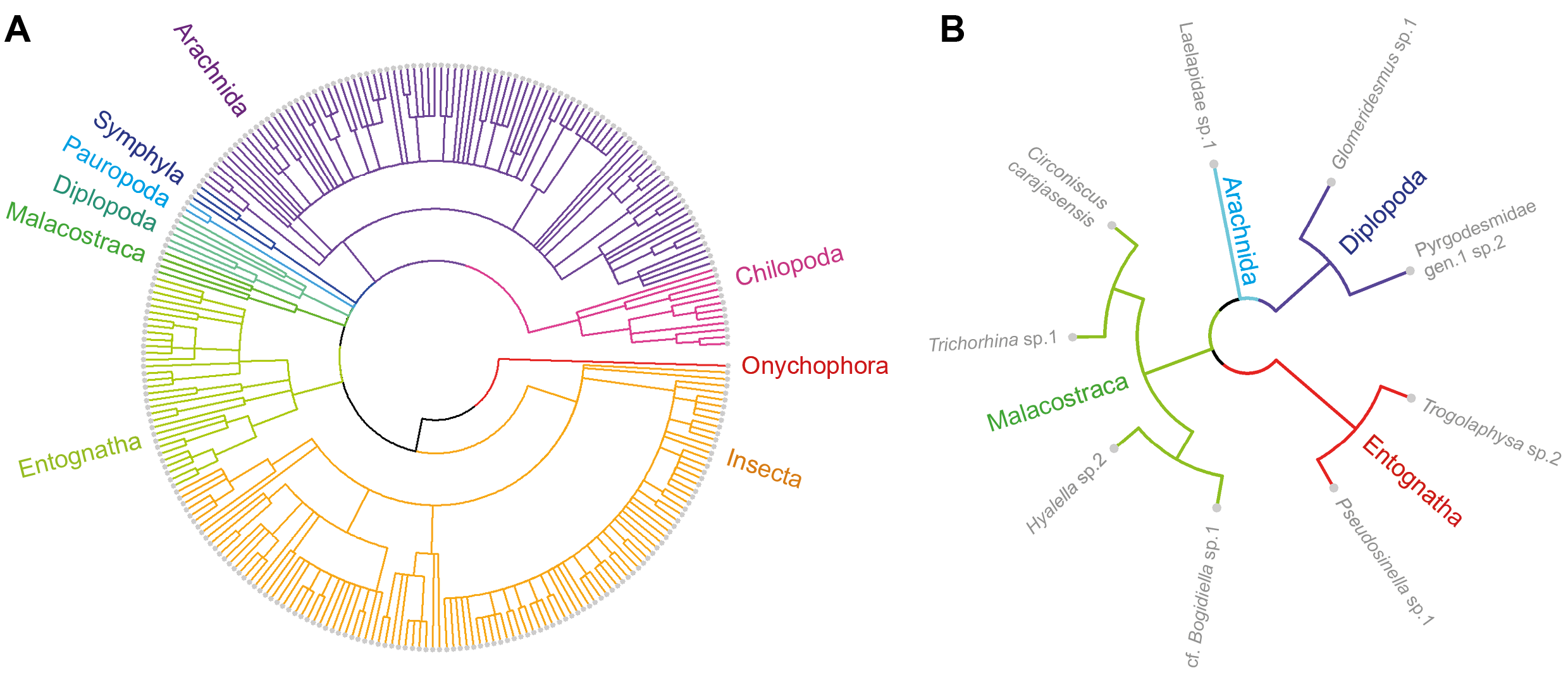

Supplement: Supplemental Information 10 — A) Considering all the cave Community (represented by 411 taxa identified at least at generic level, widely distributed in the study area). B) Only troglobites. Species groupings at class level are highlighted in the clusters. [file peerj-12-16877-s010.png]

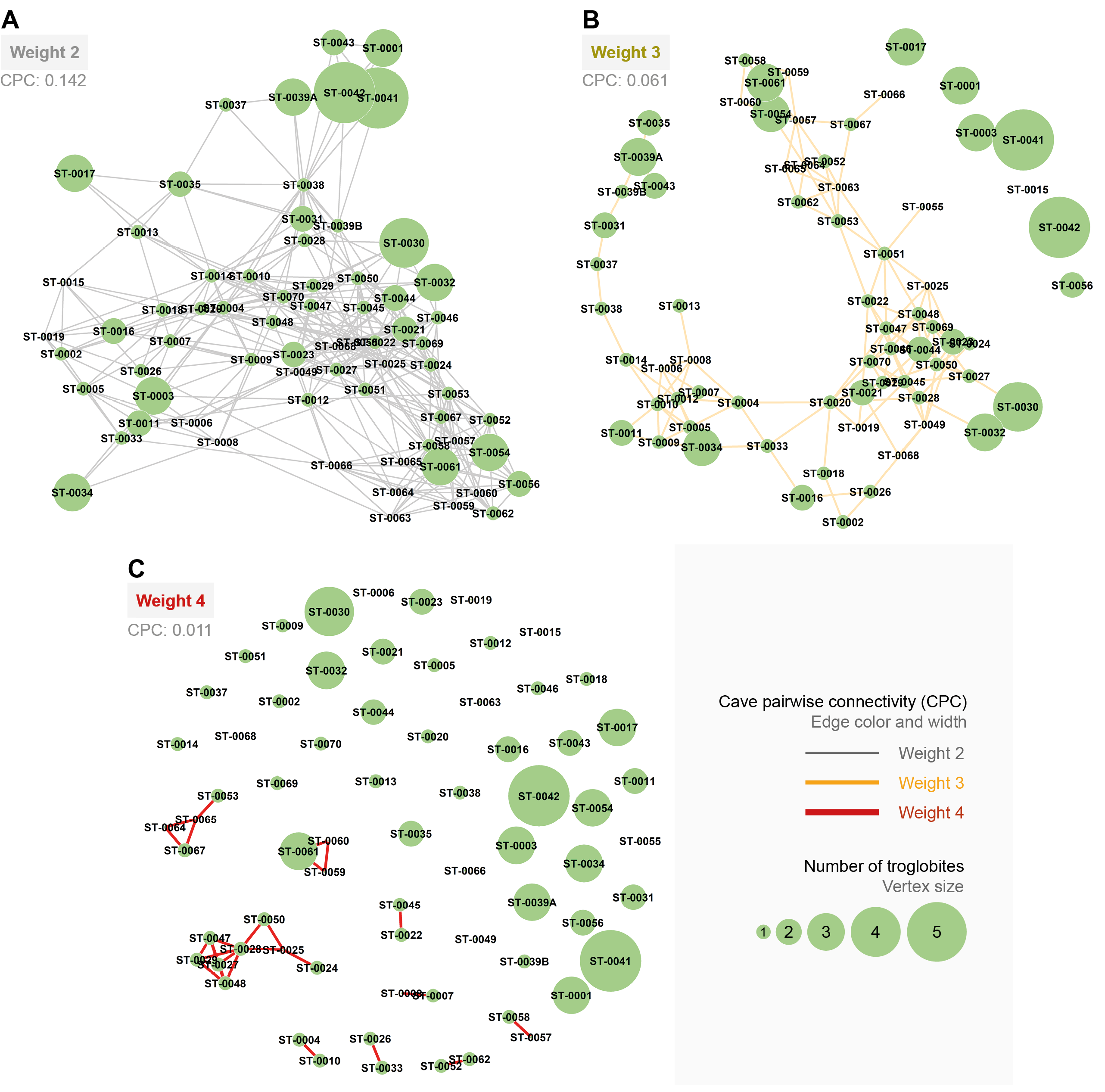

Supplement: Supplemental Information 11 — A) Weight 2 (gray), B) Weight 3 (yellow), and C) Weight 4 (red). The vertex size expresses the number of troglobitic species sampled in the cavity. [file peerj-12-16877-s011.png]
